# Backside Wear Analysis of Retrieved Acetabular Liners with a Press-Fit Locking Mechanism in Comparison to Wear Simulation In Vitro

**DOI:** 10.1155/2016/8687131

**Published:** 2016-09-19

**Authors:** Ana Laura Puente Reyna, Marcus Jäger, Thilo Floerkemeier, Sven Frecher, Karl-Stefan Delank, Christoph Schilling, Thomas M. Grupp

**Affiliations:** ^1^Aesculap AG Research and Development, 78532 Tuttlingen, Germany; ^2^Department of Orthopaedic Surgery, Physical Medicine and Rehabilitation, Ludwig Maximilians University Munich, Campus Grosshadern, 81377 Munich, Germany; ^3^Department of Orthopaedics and Trauma Surgery, University of Duisburg-Essen, 45147 Essen, Germany; ^4^Department of Orthopaedic Surgery, Hannover Medical School, 30625 Hannover, Germany; ^5^Department of Orthopaedic and Trauma Surgery, Martin Luther University Halle-Wittenberg, 06120 Halle (Saale), Germany

## Abstract

Backside wear due to micromotion and poor conformity between the liner and its titanium alloy shell may contribute to the high rates of retroacetabular osteolysis and consequent aseptic loosening. The purpose of our study was to understand the wear process on the backside of polyethylene liners from two acetabular cup systems, whose locking mechanism is based on a press-fit cone in combination with a rough titanium conical inner surface on the fixation area. A direct comparison between in vitro wear simulator tests (equivalent to 3 years of use) and retrieved liners (average 13.1 months in situ) was done in order to evaluate the backside wear characteristics and behavior of these systems. Similar wear scores between in vitro tested and retrieved liners were observed. The results showed that this locking mechanism did not significantly produce wear marks at the backside of the polyethylene liners due to micromotion. In all the analyzed liners, the most common wear modes observed were small scratches at the cranial fixation zone directly below the rough titanium inner surface of the shell. It was concluded that most of the wear marks were produced during the insertion and removal of the liner, rather than during its time in situ.

## 1. Introduction

Aseptic loosening of the implant is the main reason for revision in total hip arthroplasty (THA), with over 50% of the cases [1–3]. The main stimulator of periprosthetic osteolysis and subsequent aseptic loosening is the particulate debris generated by the wear at the articulating surface between the acetabular polyethylene liners and the femoral heads [2, 4–6]. However, there are also other sources of particulate debris generation, such as wear due to impingement, the presence of third-body particles, or micromotion between an insert and its metallic acetabular shell, also known as backside wear [7]. Hence, different implant designs and materials used in hip arthroplasty have been developed in order to decrease the polyethylene wear particle generation.

Currently, the most widely used components in THA are the modular metal-backed acetabular components. Since their development in the 1970s [8], the modular components showed some advantages such as intraoperative flexibility, multiple options for screw placement, and the opportunity to exchange the polyethylene liner during revision surgeries without removing the metallic shell. However, these types of components also brought disadvantages, like an altered stress transmission and micro- and macromotion between the liner and its metallic shell. Creep and wear on the backside of the polyethylene liner are thus generated, therefore an additional source of particulate debris that increases the risk of osteolysis and eventual aseptic loosening of the prostheses [9–11].

Particularly high backside wear has been associated with micromotion between the liner and its shell due to an unstable locking mechanism and a poor conformity between both components [11–13]. This type of wear was implicated in the high rates of retroacetabular osteolysis observed in liners that were locked to their metallic shell by means of a titanium locking ring or using a hexagonal thin polyethylene rim at the base of the liner, which fitted to a complementary groove in the metallic shell [9, 14–16]. Moreover, if there is a dissociation of the liner from the shell, the debris generated at the articulating surface can migrate between the liner and the shell and the screw holes can act as conduits for a further migration of the debris into the pelvic bone stock with the risk of inducing osteolysis [17].

Different designs have been developed in order to reduce backside wear and prevent the migration of wear debris into the acetabular bone stock [18]. These designs include improving the locking mechanism between the liner and the shell, polishing the inner surface of the shell, or sealing the screw holes with modular caps. The purpose of our study was to understand more of the wear process on the backside of polyethylene liners from two acetabular cup designs with long- and short-term clinical history, whose locking mechanism is based on a press-fit cone in combination with a rough titanium inner surface at the rim of the metallic shell. A direct comparison between in vitro tested and retrieved liners was done in order to evaluate the backside wear characteristics and behavior.

## 2. Materials and Methods

**Figure 1:**
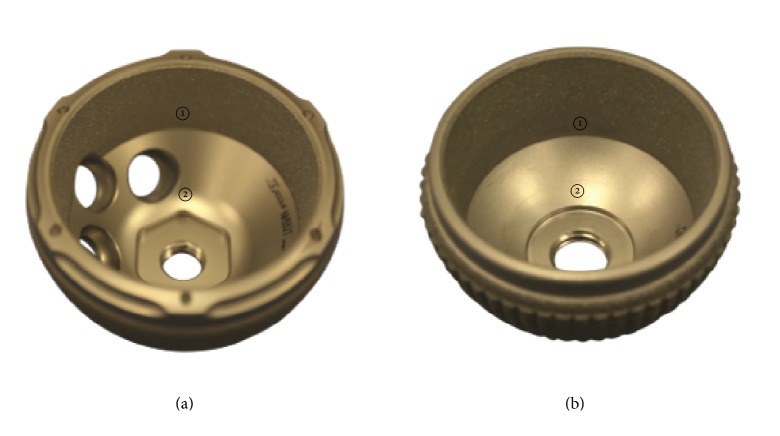
(a) P-Cup and (b) P-Fit metallic shell with grit blasted rough titanium conical inner surface at the rim ① and milled-drilled smooth surface on the concave area ②.

An optical analysis of polyethylene liners from two different cup systems (Plasmacup® and Plasmafit®, Aesculap AG, Tuttlingen, Germany) was performed (Figure 1). Both cup designs present the same locking mechanism between the liner and the shell, which is based on a press-fit cone with a large surface area and through a contact with the base of the shell, which will be achieved after the load in service. A grit blasted rough titanium inner surface (Rz = 20–32 *μ*m) along the rim of the shell intends to stabilize the liner to it. Furthermore, the conical fixation surface of the liners intends to form a seal against the migration of wear particles from the articulation joint. The screw drill holes of the Plasmacup (further referred to as P-Cup) are located in the cranial region of the shell. The Plasmafit liners (further referred to as P-Fit) analyzed did not have screw drill holes. The polyethylene liners of the two different cup systems can have either a symmetrical (Sym) or a posterior wall (PW) design. In the symmetrical design, the liners fit symmetrically in the shells, whereas the posterior wall liners contain a polyethylene hood that extends outside the shell in the luxation direction in order to increase luxation stability.

Liners from three different polyethylene materials were analyzed for backside wear. The conventional standard polyethylene liners (STD) were packed under nitrogen atmosphere and sterilized by gamma-irradiation (30 ± 2 kGy). The highly cross-linked polyethylene liners (XPE) were cross-linked by *γ*-irradiation (75 kGy) and sterilized by ethylene oxide. The highly cross-linked and Vitamin E (0.1%) blended polyethylene liners (VitE) were cross-linked by an electron beam (80 kGy) and sterilized by ethylene oxide. The polyethylene liners were tested in combination with acetabular shells made out of Ti6Al4V alloy and modular heads made out of ceramic or cobalt-chromium (Tables 1 and 2). Large femoral head and shell diameters were chosen, as these produce a high amount of wear at the articulation surface but with a low risk of luxation. Furthermore, as there is no worst case size reported for backside wear, we have chosen the most common clinically used diameters (32 and 36 mm). All the polyethylene liners for the in vitro wear tests were subjected to artificial aging according to ASTM F2003-02 at 70°C in pure oxygen at 5 bar for two weeks (Millipore Corp., 6700P05, Merck KgaA, Darmstadt, Germany). All liners were soaked prior to wear simulation in serum-based test medium until the incremental mass change over 24 h was less than 10% of the previous cumulative mass change to allow for saturated fluid absorption.

In vitro wear simulation was performed on a customized 6 + 2 (reference) stations servo hydraulic hip simulator (EndoLab GmbH, Thansau, Germany) with kinematic and load patterns according to ISO 14242-1:2012 (E). The liners were tested through 5 million cycles with a frequency of 1 Hz in a lubricant of newborn calf serum (Biochrom AG, Berlin, Germany) diluted with deionized water to achieve a target protein content of 30 g/L. The lubricant was incubated at 37°C, pH-stabilized with ethylene diamine tetraacetic acid, and replaced at intervals of 0.5 million cycles. Patricin was added to prevent fungal decay. Every 0.5 million cycles, the polyethylene liners were removed from the acetabular shell in order to perform gravimetric wear measurements and image documentation [18]. It is estimated that the 5 million cycles required in the ISO 14242-1:2012 (E) represent a mean in vivo service life of 2.9 years [19], as several studies that have estimated the gait cycles per year in patients before and after total hip or knee arthroplasty measured an average of 1.76 million gait cycles per year (range of 0.9–3.2 million gait cycles) [20–24].

Retrievals, all Plasmacup liners, were explanted during hip arthroplasty revisions in various hospitals in Germany for various reasons. P-CupD and P-CupS refer to Plasmacup DC and SC acetabular shells, which have no significant design difference. Three of the five explants that were harvested and sent to Aesculap stem from a prospective randomized study to investigate clinical and radiological differences in behavior of two different polyethylene types [25]. Between removal and optical analysis, these liners were cleaned through an ultrasonic bath in mild detergent, individually vacuum packed under nitrogen atmosphere, and stored on a freezer at −20°C. The two other liners were cleaned, individually packed under air atmosphere, and stored at room temperature. The mean survival time for all implants was 13.1 months (from 0.5 to 37 months). The liners were implanted between 2006 and 2014 and their reasons for removal were luxation (20%), infection (20%), and stem related reasons like loosening, fracture, and subsidence (60%).

**Figure 2:**
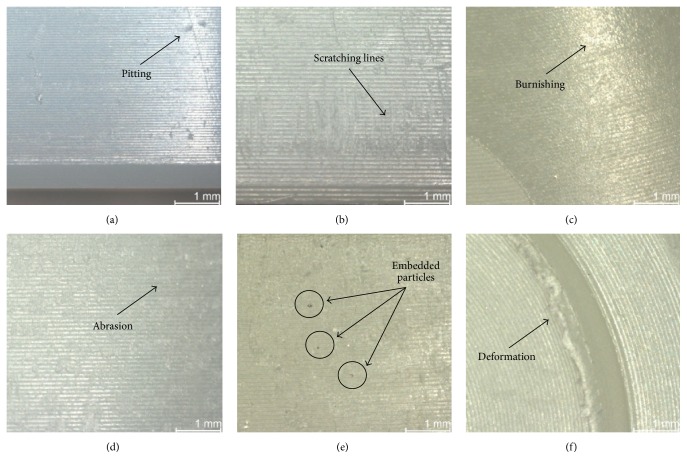
Images from each wear mode: (a) pitting, (b) scratching, (c) burnishing, (d) abrasion, (e) embedded particles, and (f) deformation. No image of delamination was taken, as this wear mode was not present in any of the inserts.

The backside surface of the acetabular liners was inspected using a stereo light microscope (Leica MZ 16, Bensheim, Germany) up to a 25x magnification. Additional images were obtained through Scanning Electron Microscope (SEM) (Zeiss EVO 50, Oberkochen, Germany) equipped with energy dispersive spectrometer (EDX) (Oxford Instruments X-Max, Wiesbaden, Germany) in order to analyze the composition of embedded particles. A semiquantitative method developed by Hood et al. [26] and modified for hip implants was used to assess the damage on the backside of the liners. Seven different modes of damage were defined (Figure 2). Deformation was used to describe the evidence of permanent deformation from the original shape due to cold flow and/or creep. Pitting described small circular indentations. Embedded particles were defined as particles embedded in the polyethylene and were recognized by the color and/or texture difference within the polyethylene surface. Scratching described straight lines that cut into the polyethylene. Burnishing described areas that had become highly polished and thus machining marks were worn off. Abrasion was defined as an area with roughened texture due to repeated rubbing. Finally, delamination described areas where a large section of polyethylene had been lost. Care was taken to differentiate the wear marks that were thought to have occurred during insertion and removal of the liner from the wear marks generated due to the liner's micromovement in service.

**Figure 3:**
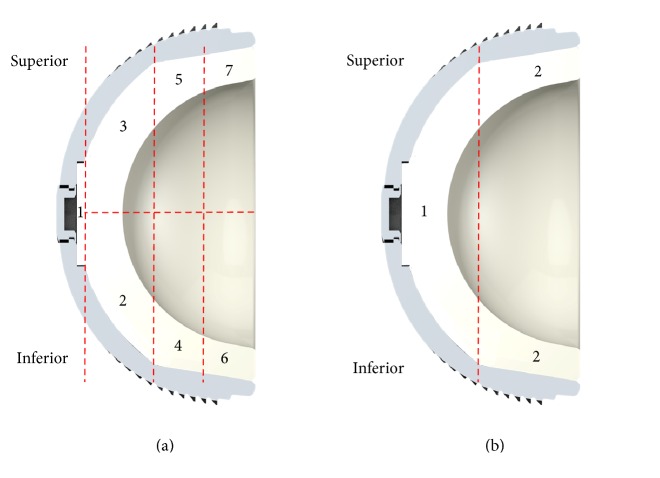
Sketch from a cross section of a P-Fit liner with its titanium alloy shell; (a) backside sections for wear analysis; (b) backside sections for screw indentation analysis.

On basis of its in situ orientation, the backside section was divided by a superior/inferior line and 7 different sections were determined (Figure 3(a)). Sections  2 and 3 correspond to the convex surface below the milled-drilled area of the shell, whereas Sections  4 to 7 correspond to the rim below the rough titanium inner surface of the shell. Damage scores for the backside surface of each liner were determined. For each section, a score between 0 and 3 was given for each of the seven damage modes, giving a maximum possible damage score of 21 per section. Following Hood's method [26], a score of 0 meant no damage; a score of 1 meant damage to less than 10% of the surface area, 2 meant damage to 10–50% of the surface area, and 3 meant that more than 50% of the area had been damaged. The grading system also combined the severity of the damage with its extent. For example, if several large scratches cover less than 50% of the section, it would be graded as 3, the same grade that would be given if small scratches cover most of the area. Each component was given a total damage score based on the sum of the scores from all its seven sections. Thus, the maximum possible damage score was 147. In case of the in vitro tested liners, a total of three liners were analyzed per group and their scores were averaged.

Moreover, the presence of creep on the backside of the liner into the screw drill holes of the metallic acetabular shell was evaluated for the P-Cup liners. Applying the grading system used by Schroder et al. [27], the liners were divided into two sections (Figure 3(b)) and each was graded according to the presence of screw holes. A grade of 0 was given if no visual evidence of creep was observed, a grade of 1 when visual evidence was observed but no palpable step could be felt, and a grade of 2 when both visual evidence and a palpable step were noted.

Two sets of observations were performed by one author (ALPR) in a time distance of one month and the scores were averaged. The intraobserver reliability of this method was between “substantial” and “almost perfect,” with kappa measures ranging between 0.72 and 0.88 for retrieved liners and between 0.69 and 0.91 for in vitro tested liners.

To differentiate between the inserts' manufacturing materials (STD versus VitE and XPE versus VitE), the metallic shell model (P-Cup versus P-Fit), and the articulating femoral heads (CoCr versus ceramic) of the in vitro tested groups, an analysis of variance was carried out (*p* = 0.05) followed by a post hoc test (Scheffe *p* = 0.05). Prior to the analysis, the normal distribution (p-p plots) and the homogeneity of variance (Levene test) were verified (Statistica 10, StatSoft Europe GmbH, Hamburg, Germany). A *p* value less than 0.05 is considered as significant.

## 3. Results

### 3.1. General Results

**Figure 4:**
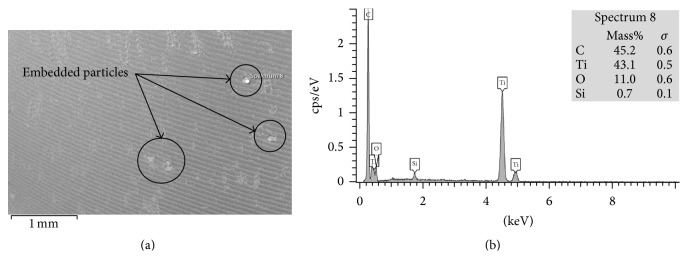
(a) SEM image with embedded particles in Section  5 from P-CupD_PW VitE_ retrieval; (b) EDX analysis of the selected particle, which apparently consists of titanium alloy. The carbon spectrum corresponds to the surrounding polymer of the liner.

The most common wear modes observed on the backside of in vitro tested and retrieved liners were scratching, abrasion, burnishing, and embedded particles (Table 3). Scratching, with an average score of 1.62 (±1.62) for in vitro tested liners and 1.36 (±0.83) for retrieved liners, had the highest score. No delamination and practically no deformation nor pitting was found in any of the in vitro tested and the retrieved liners. The highest score difference on the wear modes between the in vitro tested and the retrieved liners was found on the embedded particles, as in the in vitro tested liners just a few particles were found in comparison with the retrieved liners (score of 0.07 (±0.24) and 0.70 (±0.82), resp.). Scanning electron microscopy and EDX confirmed that the embedded particles were titanium particles (Figure 4).

### 3.2. In Vitro Wear Simulated Liners

**Figure 5:**
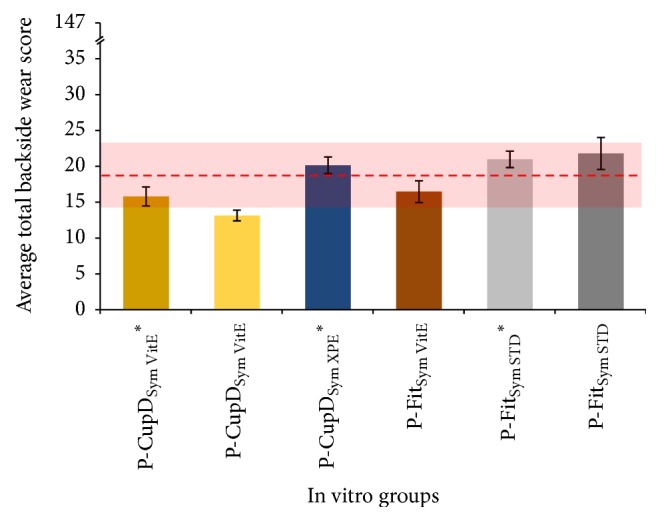
Summary of the average total backside wear score for in vitro tested liners. The dashed red line represents the average total backside wear score for the retrieved liners, while the red shadow shows the 95% confidence interval. The groups with “*∗*” were articulated against CoCr femoral heads and the others against ceramic femoral heads. Maximum total backside wear score possible = 147.

After the 5 million gait cycles' simulation, the average total backside wear score for the in vitro tested liners ranged from 13.17 (±0.75) to 21.83 (±2.23). The maximum total backside wear score possible was 147. As it can be seen in Figure 5, regardless their design, liners manufactured with STD or XPE showed a statistically higher total backside wear score compared to the liners manufactured with VitE. In case of the P-CupD liners articulated against CoCr femoral heads, the XPE group had a significantly higher average total backside wear score, with 20.17 (±0.75), in comparison with the VitE group, which had 15.83 (±1.33) (*p* = 0.0014). In case of the P-Fit liners articulated against ceramic heads, the STD group had an average total backside wear score of 21.83 (±2.23) compared to 16.50 (±1.52) of the VitE group (*p* = 0.0001).

In a direct comparison between the liners' models, when these were manufactured with VitE and articulated against ceramic femoral heads, the P-Cup group (13.17 ± 0.75) showed a significantly lower average total backside wear score than the P-Fit group (16.50 ± 1.52) (*p* = 0.0215). However, no significant difference (*p* = 0.9755) was found if the liners were manufactured with XPE or STD and articulated against CoCr femoral heads (20.17 ± 0.75 for P-Cup versus 21.00 ± 1.15 for P-Fit). Finally, no statistically significant difference in the average total backside wear score was found regarding the material of the articulating femoral head. For the P-Cup liners with VitE, the group articulated against CoCr had an average total backside wear score of 15.83 (±1.33), while the group articulated against ceramic had 13.17 (±0.75) (*p* = 0.1049). In case of the P-Fit liners with STD, the group articulated against CoCr had an average total backside wear score of 21.00 ± 1.15, while the group articulated against ceramic had 21.83 (±2.23) (*p* = 0.9755).

**Figure 6:**
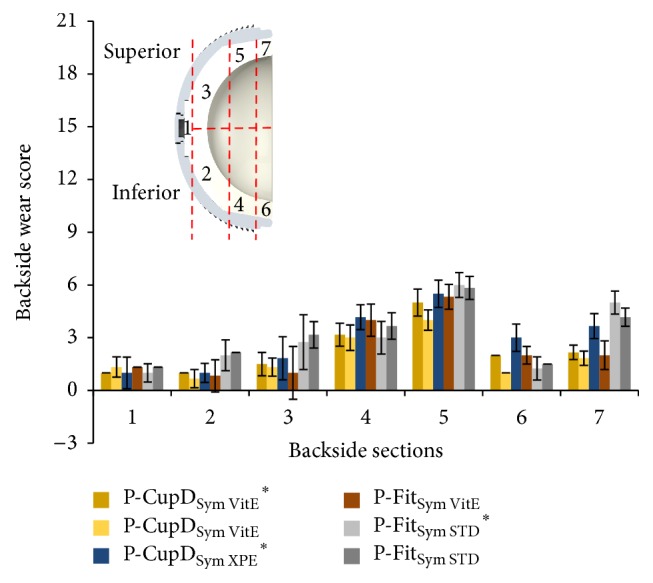
Backside wear score per backside section for in vitro wear tested liners. The groups with “*∗*” were articulated against CoCr femoral heads and the others against ceramic femoral heads. Maximum backside wear score per zone = 21.

**Figure 7:**
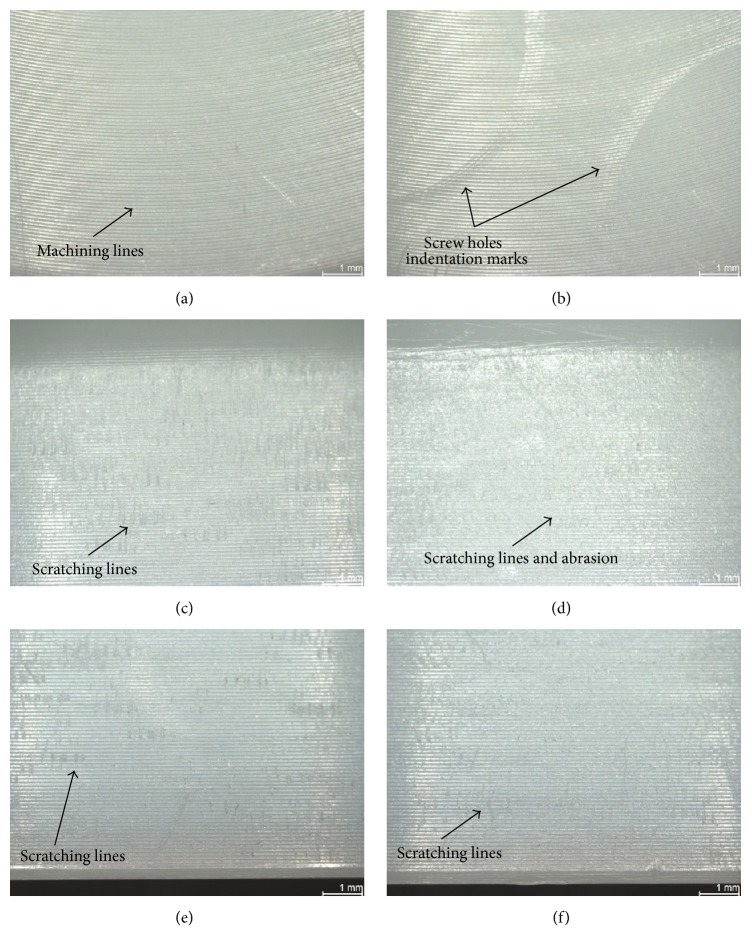
Photographs with microscope from in vitro wear tested liners. (a) P-CupD_Sym VitE_ group, Section  2, machining lines clearly visible with no wear marks; (b) P-CupD_Sym VitE_ group, Section  3, screw holes indentations marks and machining lines still visible; (c) P-CupD_Sym VitE_^*∗*^ group, Section  4, small scratching lines; (d) P-CupD_Sym VitE_^*∗*^, Section  5, scratching lines and slight abrasion; screw hole is visible; (e) P-CupD_Sym VitE_^*∗*^, Section  6, small scratching lines; (f) P-CupD_Sym VitE_^*∗*^, Section  7, small scratching lines.

**Figure 8:**
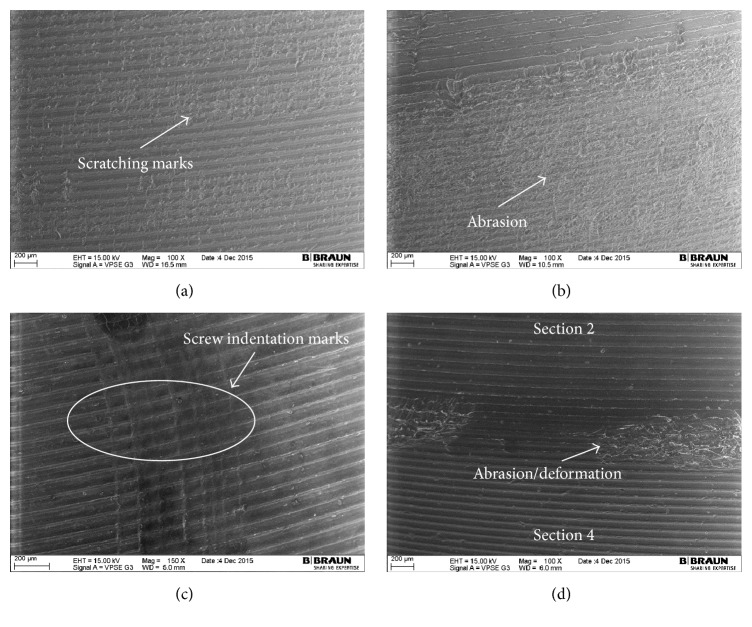
SEM images from the backside of the liners. (a) In vitro tested liners P- Fit_Sym VitE_, Section  7 with scratches and visible machining marks; (b) in vitro tested liner P-Fit_Sym VitE_, Section  5 with apparently considerable abrasion but with machining marks still visible; (c) in vitro tested liner P-CupD_Sym VitE_, Section  3 with multiple screw indentations and clear machining lines on both sides of the hole; (d) retrieval P-CupD_PW VitE_, edge between Sections  2 and 4 with small abrasion/deformation.

The most damaged areas were Sections  4 through 7 (Figure 6), corresponding to the area against the roughened titanium surface of the acetabular metallic shell, with backside wear scores between 1.00 (±0.00) and 6.00 (±0.71) from a maximum score of 21. In these sections, the mode of wear mostly observed was multiple small scratches produced by the roughened inner surface during the repeated insertion and removal of the polyethylene liner in the acetabular shell (Figures 7(c), 7(e), and 8(a)). Section  5, corresponding to the limit between the roughened and milled-drilled section of the metallic acetabular shell on the superior orientation, showed the most wear marks overall, with a backside wear score between 4.00 (±0.58) and 6.00 (±0.71). It was observed that, in this section, the multiple scratches produced abrasion of the liner and the machining marks were not more seen in some areas of the section (Figures 7(d) and 8(b)). However, it was also observed that even if a section in the superior orientation was damaged, its corresponding inferior section could be almost free of wear (Figures 7(a), 7(b), 7(e), and 7(f)). In the sections under the milled-drilled acetabular inner surface, mainly in Section  3, only a few scratches and a slight flattening/burnishing were observed, having low backside wear scores between 1.00 (±0.00) and 2.75 (±1.56).

In all the P-Cup liners, creep due to the screw holes of the metallic acetabular shell was visible but not palpable (score of 1). Near the creep produced by the screw holes, several indentations were observed due to the repeated removal every 0.5 million cycles of the liners (Figures 7(b) and 8(c)). In both the area inside and outside the screw hole creep marks, the machining marks were still clearly visible over the milled-drilled surface. In some cases, particularly on the STD and XPE liners, the machining lines appeared to be slightly flattened or burnished.

### 3.3. Retrieved Liners

**Figure 9:**
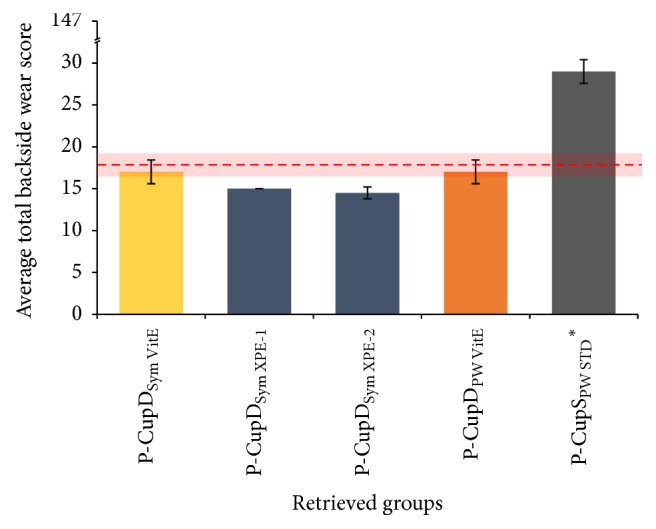
Summary of the total average wear score for retrieved liners. The dashed red line represents the total average backside wear score for the in vitro tested liners, while the red shadow shows the 95% confidence interval. The liner with “*∗*” was articulated against a CoCr femoral head; the others were articulated against ceramic femoral heads. Maximum total backside wear score possible = 147.

The average total backside wear score for the retrieved liners, whose implantation time varied from 0.5 to 37 months, ranged from 14.50 (±0.71) up to 29.00 (±1.41) (Figure 9). The maximum total backside wear score possible was 147. All the retrieved liners were P-Cup implants. In general, P-CupD liners showed less total average backside wear score, between 14.50 (±0.71) and 17.00 (±0.71), than the P-CupS liner, which had a score of 29.00 (±1.41). The two retrieved liners manufactured with Vitamin E, one P-Cup DC symmetrical and one P-Cup DC posterior wall, had a total average wear score of 17.00 (±0.71).

**Figure 10:**
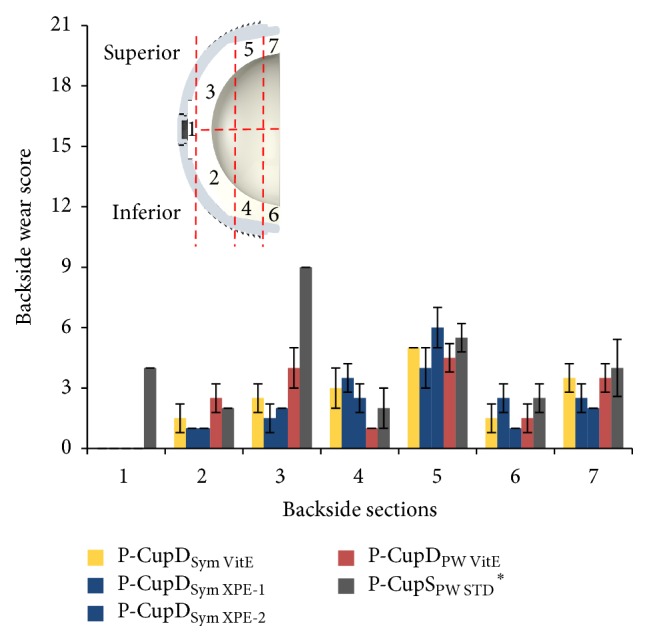
Wear score per backside section for retrieved liners. The liner with “*∗*” was articulated against a CoCr femoral head; the others were articulated against ceramic femoral heads. Maximum backside wear score per zone = 21.

**Figure 11:**
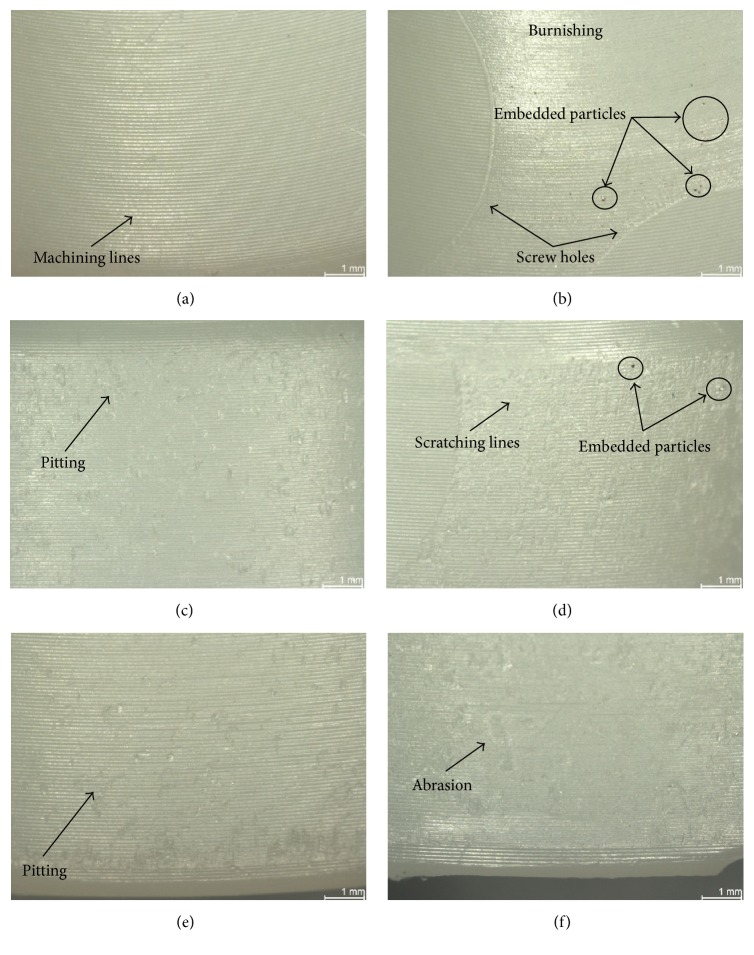
Photographs with microscope from P-CupS_PW STD_^*∗*^ retrieved liner after 37 months in situ; (a) Section  2: machining lines clearly visible with no major wear marks; (b) Section  3: screw holes visible but not palpable, with machining lines clearly visible and the periphery scratched and burnished with embedded particles; (c) Section  4: pitting and machining marks still visible; (d) Section  5: area with embedded particles and covered with scratches, machining marks partially visible; (e) Section  6: small pitting visible; (f) Section  7: area with abrasion marks.

Figure 10 shows that the P-CupD liners had their highest wear score in Section  5, with an average backside wear score between 4.00 (±1.00) and 6.00 (±1.00). In case of the P-CupS liner, the highest score was found in Section  3, a superior located section, with a score of 9 (±1.00). The high score presented in this section was due to the presence of multiple embedded particles as well as a considerable scratching, abrasion, and moderate burnishing (Figure 11). Overall, the wear modes mostly seen were small scratches produced during the insertion and removal of the liner in the metallic shell (Sections  4–7), generating some degree of abrasion on Sections  5 and 7, and embedded particles (Figures 11(d) and 11(f)).

In all the P-CupD liners, no creep due to the screw holes could be seen in the section below the milled-drilled shell, whereas, below the roughened section, the screw hole could be seen but was not palpable. Regarding the P-CupS liner, the screw holes were visible but not palpable in both sections.

## 4. Discussion

The purpose of our study was to understand more of the wear process on the backside of polyethylene liners. This was done via optical analysis of the backside of two acetabular cup designs with long- and short-term clinical history, whose locking mechanism is based on a press-fit cone in combination with a rough titanium inner surface at the rim of the metallic shell. A direct comparison between in vitro tested and retrieved liners was done in order to evaluate the backside wear characteristics and behavior. To the best of our knowledge, this is the first study to analyze backside wear on acetabular liners with this particular locking mechanism.

Because of their nature, implant retrieval analysis studies are in general limited and imperfect in study design, as they usually deal with specimens that have been removed due to a clinical failure [28]. Moreover, there is often a broad heterogeneity among the analyzed specimens, such as implant size, articulation material, implant positioning, patient loads and activity level, and time in vivo. One of the biggest limitations of the current study was the limited number of retrievals available for analysis (*n* = 5).

On the other hand, the current study had the strength that all the liners were machined from a single resin depending on their group (GUR1020 for conventional PE, GUR1020X for highly cross-linked PE, or GUR1020E for highly cross-linked and Vitamin E blended PE). Furthermore, the in vitro tested liners within each group had the same batch number and all the liners underwent the same testing and handling procedures. Besides, the in vitro tested liners were selected from batches intended for commercial sale; thus, they had the same manufacturing procedure as the retrieved liners. Moreover, the wear rates produced by the ISO hip simulator during 5 million cycles are in the clinically observed range for ceramic heads coupled with polyethylene liners [29] and represent approximately 2.9 years of in vivo service life [19–24], in comparison to the average 1.09 years of the retrieved liners (range from 2 weeks to 3 years).

Minimizing polyethylene wear is an important goal in the design of total joint replacements, as a mechanical wear of the polyethylene liner may lead to implant failure and the need for revision surgery [11]. Several locking mechanisms have been designed to prevent micromotion and backside wear, obtaining different results [8, 15, 16, 30]. The present study showed that the press-fit locking mechanism of the Plasmacup, which has proven itself successful in clinical practice since 1997 [31], and Plasmafit did not significantly influence the backside of the analyzed polyethylene liners, as most of the small wear marks were limited to the fixation area. In all the analyzed liners, the most common mode of wear observed was small scratches at the zone directly below the rough titanium inner surface of the shell. No major difference regarding the wear modes and patterns was observed among the different liner sizes. These scratches were produced during the insertion of the liner into the shell. In case of the in vitro tested liners, these were repeatedly removed and inserted every half million cycles through 5 million cycles due to the test protocol. For this reason, more scratches and several screw indentations marks (Figure 8(c)) were observed.

It could be observed that the total average backside wear score of the P-CupS_PW STD_^*∗*^ retrieval was higher than the P-CupD retrievals and in vitro tested liners. This higher score was mainly influenced by the higher scores obtained in Sections  1 and 3, which had a high amount of embedded titan particles and a higher score on scratching and burnishing. The rest of the sections had a similar score to the other liners. Wear is influenced by several factors other than a liner or shell design, such as the experience of the surgeon, method of implantation, femoral head size and cup orientation [32], and the patient gait characteristics, activity level, weight, and postoperative range of motion [33]. For these reasons, different wear scores and wear patterns could be observed even within the same liner designs. Moreover, a previous in vitro wear simulation study from Grupp et al. [18] showed that Plasmacup liners machined with conventional PE and aged for two weeks had approximately seventeen times more cumulative wear than aged liners machined with Vitamin E. Thus, the reason for the higher backside wear score from this retrieval could be attributed to the manufacturing material, longer in situ time, the patient's weight and activity level, or damage produced during the fracture of the stem.

Several facts helped to determine the micromotion between the liner and the shell. First, the small scratches produced during insertion were clearly seen and no big abrasion due to movement was observed in most of the rim area. Second, the machining marks on the convex surface beneath the milled-drilled area of the metallic shell were still clearly visible in most of the in vitro tested and retrieved liners (Figures 7(a), 8(c), 8(d), and 11(a)). Third, even though the screw drill hole edges produced indentations, the machining lines in the periphery just appeared to be flattened and were not blurred (Figures 7(b) and 8(c)). Fourth, the P-CupD_Sym XPE-1_ retrieved insert that was implanted just for two weeks had a total backside wear score similar to the rest of the retrieved P-CupD liners that were longer in situ and similar to the in vitro tested liners. This could demonstrate that most of the backside wear produced on the liners occurred during their insertion and not during the period in situ. Further studies should be done in order to quantify the backside wear produced solely during the insertion and removal of the liner. Finally, as mentioned before, the in vitro wear simulation study from Grupp et al. [18] showed a seventeen times difference in the cumulative wear between conventional PE and Vitamin E liners. However, even though our results also showed a higher backside wear score for the liners manufactured with conventional PE in comparison with those manufactured with VitE, this difference was not as high as that found in the previous study. The fact that the average total backside wear scores between the two materials were not as significantly different as the cumulative wear confirms that backside wear is produced during the insertion and removal of the liner and does not have a substantial contribution to overall wear.

The only zone where abrasion could be observed was at the edge along a small zone between the rim and the convex area (Figures 7(d), 8(b), 8(d), and 9(d)). However, a closer analysis with scanning electron microscopy (Figure 8(b)) confirmed that the machining marks were still visible. Several studies have proven through a heat-treatment of conventional PE liners that not all deep scratches necessarily mean loss of material but small deformations or cold flow, as machining lines reappear after the mentioned treatment [34–36]. Deformation of the liner at the edge between the milled-drilled and roughened sections can be expected due to the change of surface characteristics in this zone.

Moreover, it was observed that the anatomically superior located zones had the higher backside wear scores, and, in some cases, its corresponding inferior zone had a considerably lower backside wear score (Figure 11). Kawaji et al. [9] performed a retrieval analysis of polyethylene liners for backside wear and also found that the most changed surface area was the superior-anterior quadrant followed by the superior-posterior quadrant. The reason for a higher wear score in these sections is the orientation of the axial joint load transmitted to the liner. Kligman et al. [11], who performed an optical analysis on retrieved hip liners, observed creep at the superior-lateral quadrant on the convex surface of the polyethylene liner and associated this creep and its location due to the cyclical axial loading, which is transmitted mostly to the superior-lateral part of the acetabulum. This orientation was also confirmed by Bergmann et al. [37], who measured the in vivo acting loads at the hip joint in four patients and determined the contact force vector in the hip joint.

Insufficient locking mechanism, the amount of micromotion, and the metal surface finish have been well accepted factors for backside wear [14]. Several studies have shown that suboptimal conformity between the shell and the liner could be the major influence factor on liner instability causing backside wear and subsequent osteolysis [3, 12, 14, 16]. The reason for this is that synovial fluid with debris particles from the articulation surface could occupy this empty backside space and a piston pumping mechanism generated during gait could push the debris solution into the iliac bone through the screw holes and create osteolysis [10, 16].

Proven that the locking mechanism and the liner-shell connection is stable, backside wear of polyethylene liners does not substantially contribute to the overall wear rate of polyethylene liners. Using three-dimensional finite element models, Kurtz et al. [12] showed that backside linear wear rates were three orders of magnitude less than the wear rate estimates at the articulating surface. Furthermore, the wear rates between two hole and eight hole cups designs were not substantially different. In another study with a different cup design, Krieg et al. [13] showed that only 2.8% of the rate of volumetric articular wear corresponded to the rate of backside volumetric change. As Krieg's study included creep and wear for the volumetric change, this might be the reason for their higher backside wear proportion found. Moreover, the so-called “monoblock” cups, whose polyethylene liners and cups are factory-preassembled into a single solid construct, theoretically eliminate backside wear of polyethylene liners. Nevertheless, a systematic review performed by Halma et al. [3] as well as a study by González Della Valle et al. [38] showed that there was no difference in the polyethylene wear rate between monoblock and modular acetabular components at intermediate-term follow-up.

Finally, midterm clinical studies have shown very good results of the Plasmacup system, with a low revision rate due to aseptic loosening at a minimum follow-up of eight years [31, 39]. As the retrievals from the present study were at an average of 13.1 months in situ, further long-term studies with a sufficiently large number of retrievals should be performed in the future in order to analyze the backside wear behavior in the long-term as well as in vitro tests with longer testing times.

## 5. Conclusion

In general, the total average backside wear score was approximately the same for in vitro tested and retrieved liners. The same wear modes of damage and their patterns were observed in both types of liners. More importantly, our observations confirmed the low backside wear of the liners and confirmed that the wear marks were mainly initiated during their insertion and removal rather than during their time in situ. Even though retrieval analysis may show different results among the specimens and may not always coincide with in vitro tests, they still help tracking the performance of implant materials and designs. Further tests with long-term in vivo retrievals and corresponding in vitro test periods could support the results observed, as the simulation and mean retrieval times of the current study were in the short-term range.

## Figures and Tables

**Table 1:** Summary of in vitro tested implants.

In vitro group (*n* = 3 each)	Model	Polyethylene material	Femoral head	Head diameter (mm)	Shell diameter (mm)
P-CupD_Sym VitE_^*∗*^	Plasmacup DC symmetrical	VitE	CoCr	36	52
P-CupD_Sym VitE_	Plasmacup DC symmetrical	VitE	Ceramic	36	52
P-CupD_Sym XPE_^*∗*^	Plasmacup DC symmetrical	XPE	CoCr	36	52
P-Fit_Sym VitE_	Plasmafit Poly symmetrical	VitE	Ceramic	36	50
P-Fit_Sym STD_^*∗*^	Plasmafit Poly symmetrical	STD	CoCr	32	46
P-Fit_Sym STD_	Plasmafit Poly symmetrical	STD	Ceramic	32	46

**Table 2:** Summary of retrieved implants and demographic data of patients.

Retrieval	Model	Polyethylene material	Femoral head	Head diameter (mm)	Shell diameter (mm)	Time in situ (months)	Gender (age in years at revision surgery)	Weight (kg)	Reason for revision
P-CupD_Sym VitE_	Plasmacup DC symmetrical	VitE	Ceramic	32	50	11	F (74)	—	Luxation
P-CupD_Sym XPE-1_	Plasmacup DC symmetrical	XPE	Ceramic	32	56	0.5	N/A (77)	—	Infection
P-CupD_Sym XPE-2_	Plasmacup DC symmetrical	XPE	Ceramic	32	54	15	M (64)	92	Stem loosening
P-CupD_PW VitE_	Plasmacup DC posterior wall	VitE	Ceramic	32	52	2	M (69)	109	Stem subsidence
P-CupS_PW STD_^*∗*^	Plasmacup SC posterior wall	STD	CoCr	32	58	37	M (69)	110	Stem fracture

**Table 3:** Average score per wear mode of all backside sections from in vitro and retrieved liners (maximum possible score per wear mode = 3).

Wear mode	In vitro	Retrievals
Pitting	0.00 (±1.04)	0.03 (±0.17)
Scratching	1.62 (±1.62)	1.36 (±0.83)
Burnishing	0.23 (±0.47)	0.13 (±0.41)
Abrasion	0.67 (±0.95)	0.32 (±0.65)
Embedded particles	0.07 (±0.24)	0.70 (±0.82)
Deformation	0.01 (±0.11)	0.03 (±0.17)
Delamination	0.00 (±0.00)	0.00 (±0.00)

## References

[1] Sadoghi P., Liebensteiner M., Agreiter M., Leithner A., Böhler N., Labek G. (2013). Revision surgery after total joint arthroplasty: a complication-based analysis using worldwide arthroplasty registers. *Journal of Arthroplasty*.

[2] Glyn-Jones S., Thomas G. E. R., Garfjeld-Roberts P. (2014). The John Charnley Award: highly crosslinked polyethylene in total hip arthroplasty decreases long-term wear: a double-blind randomized trial. *Clinical Orthopaedics and Related Research*.

[3] Halma J. J., Vogely H. C., Dhert W. J., Van Gaalen S. M., De Gast A. (2013). Do monoblock cups improve survivorship, decrease wear, or reduce osteolysis in uncemented total hip arthroplasty?. *Clinical Orthopaedics and Related Research*.

[4] Amstutz H. C., Campbell P., Kossovsky N., Clarke I. C. (1992). Mechanism and clinical significance of wear debris-induced osteolysis. *Clinical Orthopaedics and Related Research*.

[5] Revell P. A., Al-Saffar N., Kobayashi A. (1997). Biological reaction to debris in relation to joint prostheses. *Proceedings of the Institution of Mechanical Engineers H*.

[6] Kurtz S. M., Gawel H. A., Patel J. D. (2011). History and systematic review of wear and osteolysis outcomes for first-generation highly crosslinked polyethylene. *Clinical Orthopaedics and Related Research*.

[7] Wright T. M., Goodman S. B. (2001). What are the wear mechanisms and what controls them?. *Implant Wear in Total Joint Replacement: Clinical and Biologic Issues, Material and Design Considerations*.

[8] Williams V. G., Whiteside L. A., White S. E., McCarthy D. S. (1997). Fixation of ultrahigh-molecular-weight polyethylene liners to metal- backed acetabular cups. *The Journal of Arthroplasty*.

[9] Kawaji H., Koistinen A., Korhonen R. (2014). Back-side wear in HexLoc cups clinico-radiological, immunohistopathological, finite element, and retrieval analysis studies. *Journal of Long-Term Effects of Medical Implants*.

[10] Gonzalez Della Valle A., Sculco T. P. (2006). Complications of acetabular modularity. *Business Briefing: US Orhopaedics Review*.

[11] Kligman M., Furman B. D., Padgett D. E., Wright T. M. (2007). Impingement contributes to backside wear and screw-metallic shell fretting in modular acetabular cups. *Journal of Arthroplasty*.

[12] Kurtz S. M., Ochia J. A., Hovey C. B., White C. Frontside vs Backside wear in an acetabular component with multiple screw holes.

[13] Krieg A. H., Speth B. M., Ochsner P. E. (2009). Backside volumetric change in the polyethylene of uncemented acetabular components. *The Journal of Bone & Joint Surgery—British Volume*.

[14] Nieuwenhuis J. J., Malefijt J. D. W., Hendriks J. C., Gosens T., Bonnet M. (2005). Unsatisfactory results with the cementless Omnifit acetabular component due to polyethylene and severe osteolysis. *Acta Orthopaedica Belgica*.

[15] Noble P. C., Durrani S. K., Usrey M. M., Mathis K. B., Bardakos N. V. (2012). Constrained cups appear incapable of meeting the demands of revision THA. *Clinical Orthopaedics and Related Research*.

[16] Powers C. C., Ho H., Beykirch S. E. (2010). A comparison of a second- and a third-generation modular cup design. *The Journal of Arthroplasty*.

[17] Khalily C., Tanner M. G., Williams V. G., Whiteside L. A. (1998). Effect of locking mechanism on fluid and particle flow through modular acetabular components. *The Journal of Arthroplasty*.

[18] Grupp T. M., Holderied M., Mulliez M. A. (2014). Biotribology of a vitamin E-stabilized polyethylene for hip arthroplasty—influence of artificial ageing and third-body particles on wear. *Acta Biomaterialia*.

[19] Schwiesau J., Schilling C., Kaddick C. (2013). Definition and evaluation of testing scenarios for knee wear simulation under conditions of highly demanding daily activities. *Medical Engineering & Physics*.

[20] Goldsmith A. A. J., Dowson D., Wroblewski B. M. (2001). Comparative study of the activity of total hip arthroplasty patients and normal subjects. *The Journal of Arthroplasty*.

[21] Seedhom B. B., Wallbridge N. C. (1985). Walking activities and wear of prostheses. *Annals of the Rheumatic Diseases*.

[22] Silva M., Shepherd E. F., Jackson W. O., Dorey F. J., Schmalzried T. P. (2002). Average patient walking activity approaches 2 million cycles per year: pedometers under-record walking activity. *The Journal of Arthroplasty*.

[23] Schmalzried T. P., Szuszczewicz E. S., Northfield M. R. (1998). Quantitative assessment of walking activity after total hip or knee replacement. *The Journal of Bone & Joint Surgery—American Volume*.

[24] Seedhom B. B., Dowson D., Wright V. (1973). Wear of solid phase formed high density polyethylene in relation to the life of artificial hips and knees. *Wear*.

[25] Longterm-Evaluation of Vitelene® against Standard (VITAS) https://www.clinicaltrials.gov/ct2/show/NCT01713062?term=VITAS+Aesculap&rank=1.

[26] Hood R. W., Wright T. M., Burstein A. H. (1983). Retrieval analysis of total knee prostheses: a method and its application to 48 total condylar prostheses. *Journal of Biomedical Materials Research*.

[27] Schroder D. T., Kelly N. H., Wright T. M., Parks M. L. (2011). Retrieved highly crosslinked UHMWPE acetabular liners have similar wear damage as conventional UHMWPE. *Clinical Orthopaedics and Related Research*.

[28] French K., Moore R., Gawel H. (2012). Retrieval analysis of Harris-Galante I and II acetabular liners in situ for more than 10 years. *Acta Orthopaedica*.

[29] Kaddick C., Wimmer M. A. (2001). Hip simulator wear testing according to the newly introduced standard ISO 14242. *Proceedings of the Institution of Mechanical Engineers, Part H: Journal of Engineering in Medicine*.

[30] Corten K., McCalden R. W., Teo Y., Charron K. D., MacDonald S. J., Bourne R. B. (2011). Midterm results of 506 solid trispiked reflection cementless acetabular components for primary total hip arthroplasty. *Journal of Arthroplasty*.

[31] Ochs U., Ilchmann T., Ochs B. G. (2007). EBRA migration patterns of the plasmacup with ceramic or polyethylene inserts: a randomised study. *Zeitschrift für Orthopädie und Unfallchirurgie*.

[32] Heiner A. D., Kruger K. M., Tikekar N. M., Callaghan J. J., Lannutti J. J., Brown T. D. (2015). THA retrievals: the need to mark the anatomic orientation of the femoral head. *The Journal of Arthroplasty*.

[33] Yamaguchi M., Hashimoto Y., Akisue T., Bauer T. W. (1999). Polyethylene wear vector in vivo: a three-dimensional analysis using retrieved acetabular components and radiographs. *Journal of Orthopaedic Research*.

[34] Knahr K., Pospischill M., Köttig P., Schneider W., Plenk H. (2007). Retrieval analyses of highly cross-linked polyethylene acetabular liners four and five years after implantation. *The Journal of Bone & Joint Surgery—British Volume*.

[35] Muratoglu O. K., Greenbaum E. S., Bragdon C. R., Jasty M., Freiberg A. A., Harris W. H. (2004). Surface analysis of early retrieved acetabular polyethylene liners: a comparison of conventional and highly crosslinked polyethylenes. *Journal of Arthroplasty*.

[36] Schneider W., Köttig P. Analysis of early retrieved acetabualar cups of highly crosslinked polyethylene.

[37] Bergmann G., Deuretzbacher G., Heller M. (2001). Hip contact forces and gait patterns from routine activities. *Journal of Biomechanics*.

[38] González Della Valle A., Su E., Zoppi A., Sculco T. P., Salvati E. A. (2004). Wear and periprosthetic osteolysis in a match-paired study of modular and nonmodular uncemented acetabular cups. *The Journal of Arthroplasty*.

[39] Lakemeier S., Aurand G., Timmesfeld N., Heyse T. J., Fuchs-Winkelmann S., Schofer M. D. (2010). Results of the cementless Plasmacup in revision total hip arthroplasty: a retrospective study of 72 cases with an average follow-up of eight years. *BMC Musculoskeletal Disorders*.

